# Machine learning prediction of gestational age from metabolic screening markers resistant to ambient temperature transportation: Facilitating use of this technology in low resource settings of South Asia and East Africa

**DOI:** 10.7189/jogh.12.04021

**Published:** 2022-04-23

**Authors:** Sunil Sazawal, Sayan Das, Kelli K Ryckman, Rasheda Khanam, Imran Nisar, Saikat Deb, Elizabeth A Jasper, Sayedur Rahman, Usma Mehmood, Arup Dutta, Nabidul Haque Chowdhury, Amina Barkat, Harshita Mittal, Salahuddin Ahmed, Farah Khalid, Said Mohammed Ali, Rubhana Raqib, Muhammad Ilyas, Ambreen Nizar, Alexander Manu, Donna Russell, Sachiyo Yoshida, Abdullah H Baqui, Fyezah Jehan, Usha Dhingra, Rajiv Bahl

**Affiliations:** 1 Center for Public Health Kinetics, New Delhi, India; 2 Public Health Laboratory-IDC, Chake Chake, Tanzania; 3 University of Iowa, Iowa City, Iowa, USA; 4 Johns Hopkins Bloomberg School of Public Health, Baltimore, Maryland, USA; 5 Aga Khan University, Karachi, Pakistan; 6 Projahnmo Research Foundation, Dhaka, Bangladesh; 7 International Center for Diarrheal Disease Research, Dhaka, Bangladesh; 8 Department of Maternal, Newborn, Child and Adolescent Health, and Ageing, Geneva, Switzerland; 9 University of California, Seattle, Washington, USA

## Abstract

**Background:**

Knowledge of gestational age is critical for guiding preterm neonatal care. In the last decade, metabolic gestational dating approaches emerged in response to a global health need; because in most of the developing world, accurate antenatal gestational age estimates are not feasible. These methods initially developed in North America have now been externally validated in two studies in developing countries, however, require shipment of samples at sub-zero temperature.

**Methods:**

A subset of 330 pairs of heel prick dried blood spot samples were shipped on dry ice and in ambient temperature from field sites in Tanzania, Bangladesh and Pakistan to laboratory in Iowa (USA). We evaluated impact on recovery of analytes of shipment temperature, developed and evaluated models for predicting gestational age using a limited set of metabolic screening analytes after excluding 17 analytes that were impacted by shipment conditions of a total of 44 analytes.

**Results:**

With the machine learning model using all the analytes, samples shipped in dry ice yielded a Root Mean Square Error (RMSE) of 1.19 weeks compared to 1.58 weeks for samples shipped in ambient temperature. Out of the 44 screening analytes, recovery of 17 analytes was significantly different between the two shipment methods and these were excluded from further machine learning model development. The final model, restricted to stable analytes provided a RMSE of 1.24 (95% confidence interval (CI) = 1.10-1.37) weeks for samples shipped on dry ice and RMSE of 1.28 (95% CI = 1.15-1.39) for samples shipped at ambient temperature. Analysis for discriminating preterm births (gestational age <37 weeks), yielded an area under curve (AUC) of 0.76 (95% CI = 0.71-0.81) for samples shipped on dry ice and AUC of 0.73 (95% CI = 0.67-0.78) for samples shipped in ambient temperature.

**Conclusions:**

In this study, we demonstrate that machine learning algorithms developed using a sub-set of newborn screening analytes which are not sensitive to shipment at ambient temperature, can accurately provide estimates of gestational age comparable to those from published regression models from North America using all analytes. If validated in larger samples especially with more newborns <34 weeks, this technology could substantially facilitate implementation in LMICs.

Preterm birth is a leading cause of neonatal morbidity and mortality worldwide, with more than 95% of its global burden being contributed by low- and middle-income countries (LMICs) [1]. Knowledge of gestational age is critical for guiding preterm neonatal care as well as quantifying its burden, thereby enabling planning and program evaluation.

In most LMICs settings where maternal access to ultrasound dating in early pregnancy is limited; measures such as last menstrual period, fundal height, or examination of the newborn are relied on for gestational age estimation [2-4]. Gestational dating based on knowledge of last menstrual period has been shown to be unreliable in these settings even in the best hands [5-7]. Newborn assessments for gestational age determination are subject to high inter-user variability and are often imprecise [2]. Therefore, current methods for estimating the burden of preterm births are handicapped [8-12] and the need for newer methods has been recognized in global health [13]. Circulating newborn metabolites are affected by gestational age, which is therefore also considered in the interpretation of newborn screening analysis [14-16].

Algorithms developed in North American settings, deriving gestational age (GA) estimates through the biochemical analysis of newborn dried blood spots (DBS), have been shown to provide accurate estimates to within 2 weeks of best obstetric estimate [3]. One of these models, based on conventional multivariable linear and logistic regression methods, have been internally validated in US population [17], and externally validated in Alliance for Maternal and Newborn Health Improvement (AMANHI) LMICs cohort, demonstrating satisfactory performance [18]. We have further demonstrated improvement in validity with an error of around 1 week by using machine learning models [19]. During external validation however, we did recognize the need for temperature control if the samples are to be shipped to a central laboratory within or outside the country (which would be most often the case if this method was to be used in LMIC settings).

Many biomarkers used for newborn screening are susceptible to heat and humidity [20,21]. Acylcarnitines have been shown to hydrolyze to free carnitines and corresponding fatty acids if stored for prolonged periods (>14 days) at room temperature [21]. Limited evidence is currently available regarding the short-term stability of amino acids and acylcarnitines in DBS [22,23] which impacts requirements for shipment of samples.

Therefore, in a pilot study we investigated the difference in the recovery of the analytes in paired samples shipped at ambient temperature and on dry ice, from LMICs sites to IOWA (USA) for tandem mass spectrometric (TMS) analysis. Realizing the loss of recovery, our published external validations consequently used only DBS shipped on dry ice [18].

We now use the pilot study data to report the impact on recovery by ambient temperature shipment. We also investigated models using machine learning with a restricted set of analytes, resistant to temperature effects. In this non-interventional, international validation study; we report changes in recovery of analytes and the accuracy of gestational age estimation comparing two shipment methods, and results from machine learning models using analytes resistant to temperature change, compared to those obtained by conventional methods using all analytes [18].

## METHODS

### Study population

This study was undertaken using data from the AMANHI, all children thrive (ACT), community based, prospective pregnancy and newborn cohorts from Pemba (Tanzania), Sylhet (Bangladesh) and Karachi (Pakistan). The rationale for these cohorts and associated biobank, procedures, and cohort characteristics have been described elsewhere [24]. One of the objectives of the AMANHI study was to develop and validate programmatically feasible approaches to accurately assess the gestational age of babies after they are born. Briefly, women were enrolled in early pregnancy and followed through delivery and the postpartum period. GA was established by ultrasonography at screening using the fetal crown rump length (if <14 weeks gestation) or biparietal diameter and femur length (if ≥14 weeks) [25,26]. All fetal biometry measurements were measured twice and then averaged for gestational age calculations [27,28]. Birth weight (5g sensitivity) was measured using standard newborn weighing scale (SECA corporation, Columbia, MD, USA).

### Informed consent and ethical approval

All study protocols for AMANHI cohorts were approved by ethical review committees of the World Health Organization (WHO) and appropriate institutional review board in each of the participating sites. Additionally, institutional/local sample utilization committees approved shipment of samples to Iowa for metabolic screening assay. Mothers were asked for additional informed consent before obtaining a heel prick from the baby.

### Sample collection and processing

**Figure 1:**
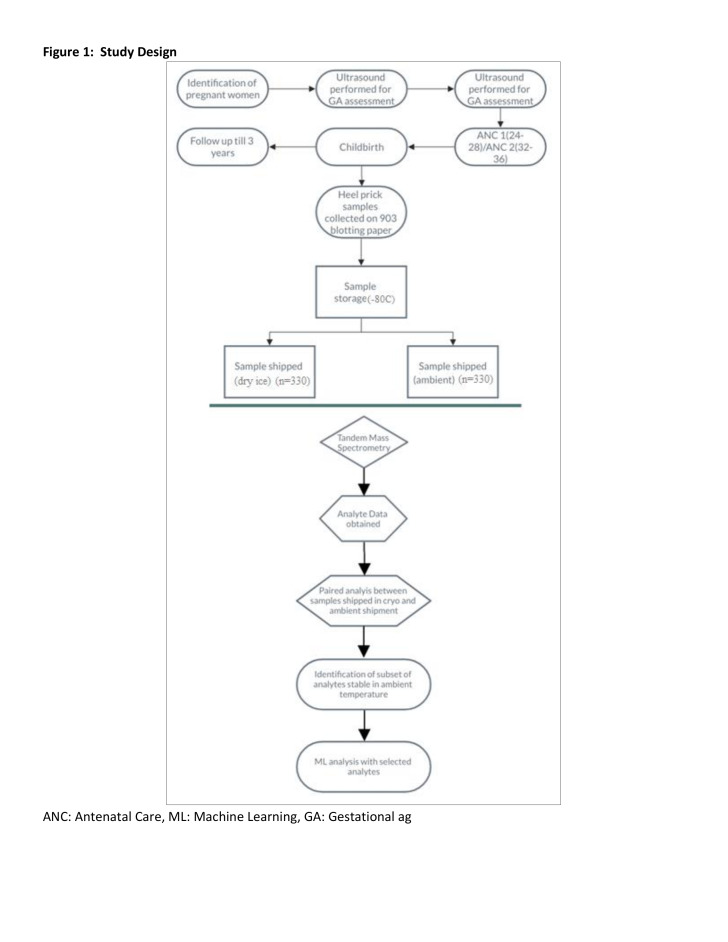
Study design. ANC – antenatal care, ML – machine learning, GA – gestational age.

The metabolic screening data from 330 samples used for this analysis was a subset of 1318 individual samples set generated as part of the AMANHI collaboration with Department of Epidemiology, College of Public Health, University of Iowa, for evaluating external validity of the GA estimation methods, developed based on Iowa samples [17,18]. Heel prick blood spots were obtained on a protein saver card (Whatman^R^ 903, GE Healthcare, USA), within 24-72 hours of birth from newborns as per standard procedures. The DBS cards were air-dried and stored in air-tight zip-lock bags with desiccant at -80°C and shipped in dry ice to the State Hygienic Laboratory, Ankeny, Iowa, USA at regular intervals (ensuring processing before potency window). For the present analysis, a small subset of 330 cards were split into two, one of them was shipped in dry ice with other cards while the other set was shipped in ambient temperature (**Figure 1**). All the metabolites which included amino acids, acylcarnitines, enzymes and hormones were analyzed using tandem mass spectrometry [17]. Only singleton births were included in the final analysis since analyte values are associated with birth status [29].

### Evaluating impact of shipping temperature on analyte recovery

**Figure 2:**
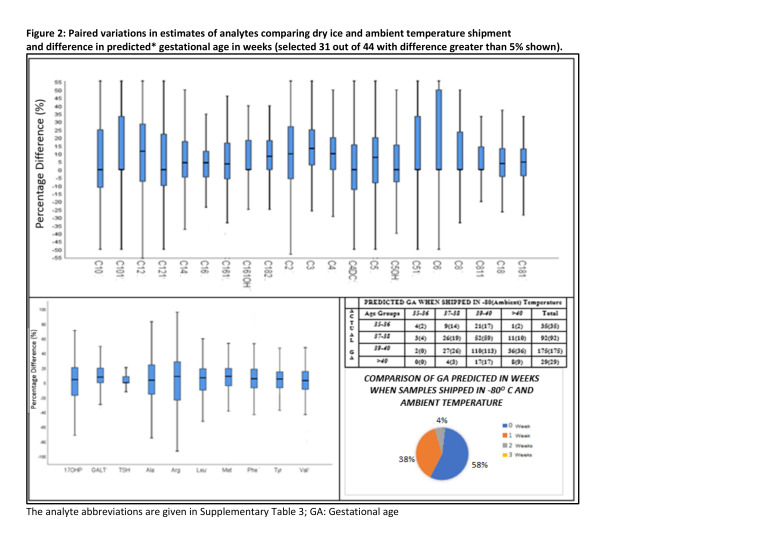
Paired variations in estimates of analytes comparing dry ice and ambient temperature shipment and difference in predicted gestational age in weeks (selected 31 out of 44 with difference greater than 5% shown). The analyte abbreviations are given in Table S3 in the **Online Supplementary Document**; GA – gestational age.

Baseline characteristics of the 330 children contributing the samples provided in **Table 1**, were evaluated against 1318 children [19], from which this sample was sub-selected. Percentage difference for each of the metabolites, between samples shipped in dry ice and ambient temperature were calculated across all the samples (**Figure 2**).

**Table 1:** Cohort characteristics of infants included in the metabolic screening study

Heel prick samples	Total cohort (n = 330)
Gender:	
Male	174 (52.7%)
Female	156 (47.3%)
Gestational age (by ultrasound at <20 weeks) mean ± SD:	38.53 ± 1.68
≥37 weeks	298 (88.4%)
<37 weeks	32 (11.6%)
Birthweight (mean ± SD):	3037.21+601.67
Birth weight category, n (%):	
<2500 g	49 (15.1%)
≥2500 g	281 (84.9%)
Multiple birth status	5 (1.5%)
Newborn sample collected (hrs) mean ± SD	49.0 ± 16.2

SD – standard deviation

### Construction and representativeness testing of simulated data set to parent validation data

For the machine learning step, to compensate for the small sample size, a simulated data set with bootstrapping was generated using the data set from 330 samples shipped on dry ice, using a python package SimPy. A normal probability distribution (*dnorm*) was used so that the means and standard deviation for the vector points remained consistent. To evaluate if the 330 selected samples and their simulated data were in fact an unbiased representation of the parent data set used in the validation exercise [18,19], we used a published machine learning model to predict the GA in the simulated data set, and compared the results with those, obtained from 1283 participants in published validation study [19]. Stacked percentage and distribution plots were made to compare the predictability. Root mean square error (RMSE) and mean absolute error (MAE) were estimated and compared to the RMSE and MAE values obtained previously.

### Machine learning algorithm for all analytes

In the first step the simulated data set from 330 samples shipped on dry ice was divided into training and test data sets using R coding. Equal number of samples was assigned to the test and training data set randomly. Sklearn. ensemble (Random Forest Regressor package [30]), a Python module was used for running the RF regressor. This selection was made with replacement. A “K-fold validation technique” was used to make the model more robust. The number of K was denoted as 10 and was repeated 3 times. NumPy, Scipy and Pandas were used as python dependencies for running the module. The trained model from this training data set using all analytes, was utilized to estimate the gestational age in two test-data sets; a) 330 samples with analyte values from DBS shipped on dry ice and b) 330 samples with analyte values from DBS shipped in ambient temperature.

### Performance metrics

The fitness of the algorithm was accessed using Root mean square error (RMSE) and mean absolute error (MAE). The RMSE of a predicted model with respect to the estimated variable *x_model_* is defined as the square root of the mean squared error [31].



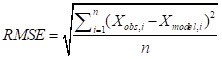



Where, *x_obs_* is observed values, *x_model_* is modelled values at time i.

Mean absolute error (MAE) has been calculated as:



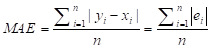



where *x_i_* is the prediction and *y_i_* is the true value.

### Confidence intervals for RMSE and MAE

Computation of 95% confidence interval for RMSE, MAE values were estimated using bootstrapped procedures [32-34] (Python package, bootstrapped 0.0.2) with a fixed seed (t) number of 1 using boot and metrics packages in R.

### Receiver Operator Characteristic (ROC) analysis for evaluating discriminatory ability of the ML based GA

For ROC analysis we used Stata 16.1 (StataCorp LLC, Texas USA) and Medcalc (MedCalc Software Ltd Belgium). Generation of ROC curve and AUC estimation was performed and interpreted using standard methods. We estimated Youden index J [35,36]: 

*J* *=* *max* {*sensitivityf* [*c*] *+* *specificityf* [*c*] *–* 1}

where c ranges over all possible criterion values. Graphically, J is the maximum vertical distance between the ROC curve and the diagonal line [37]. Bootstrapped 95% CI for Youden index and its corresponding criterion value were estimated. 95% CI for sensitivities and specificities were also estimated for a range of fixed and pre-specified sensitivities/specificities and 95% CI estimated using bootstrapping [38]. Comparison of ROC curves estimating difference, confidence interval and *P* value were also performed using bootstrap methods [39,40]. For the Bootstrap estimation, a fixed seed was used to enable replication of the analysis.

### Machine learning algorithm for restricted analytes

Based on literature review (12 metabolites) and comparison of paired percentage change between the sample shipped on dry ice and that shipped in ambient temperature for every metabolite, a total of 17 metabolites (Table S2 in the **Online Supplementary Document**) were determined to be prone to temperature effects (significant differences in change of means) [41] and excluded in second round of machine learning model development. The selected analyte variables were deleted from the simulated data set as well as the two test data sets to produce restricted analyte data sets. As a second step analysis, all the steps of the first step, training and testing were repeated with these restricted data sets. Performance metrics were compared between the predicted values of step 1 and step 2.

## RESULTS

The baseline characteristics of the 330-subsample used for this analysis, out of 1318 samples from Tanzania, Bangladesh and Pakistan used for the AMANHI/ACT validation [18] were comparable (**Table 1**) with 11.6% preterm and 15.1% low birth weight babies.

### Impact of shipment temperature on recovery of analytes

Box and whisker plots showing percentage differences in recovery between ambient temperature and dry ice shipment of 44 analytes are provided in **Figure 2**. Of the 44 analytes 17 were found to be temperature sensitive and had a significant percentage change by two shipment methods (Table S2 in the **Online Supplementary Document**). These 17 were then excluded from the restricted analyte analysis. Impact of this difference on predicted GA using the linear regression model [17], is provided in **Figure 2**, with no difference in 58% children, and 1- and 2-weeks difference in 38% and 4% respectively. RMSE was observed to be 1.6 weeks for samples shipped in dry ice while it was 2.28 weeks for samples transported at ambient temperature.

### Comparing simulated training data set (from 330 subsamples) to parent data set

When the published ML model [19] was used to predict GA in the simulated data set, a similar distribution pattern of predicted GA was observed comparing the original and the simulated database from the sub-sample (Figure S1 in the **Online Supplementary Document**). The simulated data set from 330 samples, with samples shipped on dry ice resulted in RMSE of 1.07 (95% confidence interval (CI) = 0.96-1.21) as compared to RMSE of 1.02 (95% CI = 0.91-1.14) for the parent data set (1318 children shipped and stored at -80°C and shipped on dry ice.) [19]. A similar result was obtained in terms of MAE (0.81 vs 0.76) (Table S1 in the **Online Supplementary Document**). These data provided evidence that sub-sample in this analysis was an unbiased estimator of the overall sample of 1318 children.

### Machine learning analysis step-1 comparing two shipment methods using all analytes

**Figure 3:**
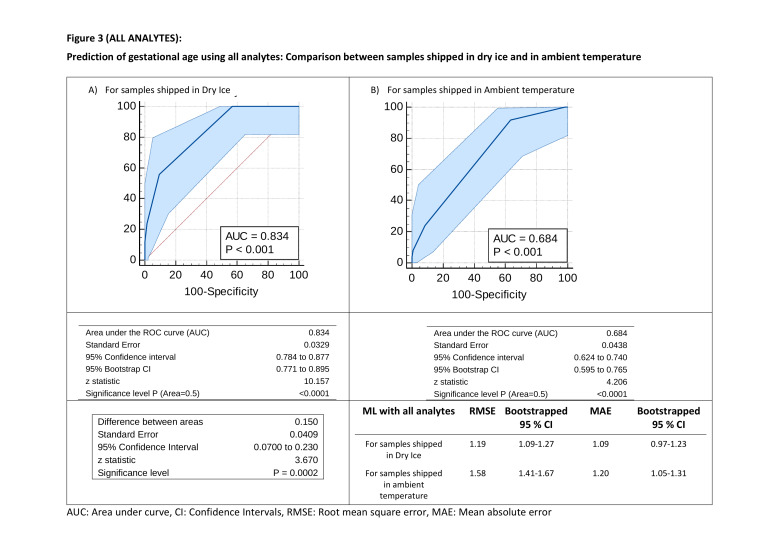
All analytes. Prediction of gestational age using all analytes: Comparison between samples shipped in dry ice and in ambient temperature. AUC – area under curve, CI – confidence intervals, RMSE – root mean square error, MAE – mean absolute error.

The step 1 analysis implementing training from simulated data onto testing the samples with two shipment methods indicated an adverse impact similar to that observed with regression analysis (**Figure 3**). RMSE values were 1.19 vs 1.58 and MAE of 1.09 vs 1.20 weeks between samples shipped on dry ice and in ambient temperature respectively. For discriminatory ability of identifying preterm births among samples shipped in dry ice including all analytes, this analysis provided AUC of 0.834 (95% CI = 0.77-0.90; *P* < 0.001) which decreased significantly with the samples that were shipped in ambient temperature (difference in AUC = 0.15; 95% CI = 0.0700 to 0.230; *P* = 0.0002) (**Figure 3**).

### Machine learning analysis step-2 comparing two shipment methods using restricted analytes

**Figure 4:**
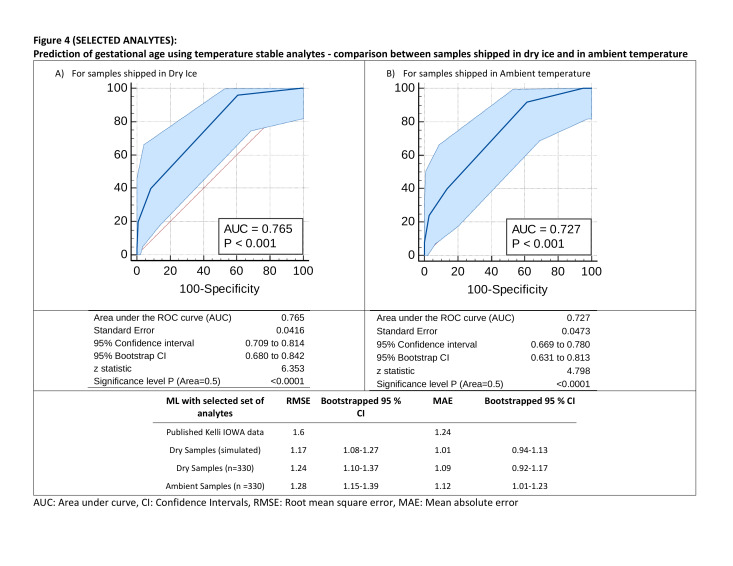
Selected analytes. Prediction of gestational age using temperature stable analytes - comparison between samples shipped in dry ice and in ambient temperature. AUC – area under curve, CI – confidence intervals, RMSE – root mean square error, MAE – mean absolute error

During the training, machine model with reduced number of analytes estimated gestation age had a RMSE of 1.17 weeks and MAE of 1.01 on the 50% simulated training data set. When the model was tested on the two paired shipment method data, RMSE values for samples shipped in dry ice and ambient temperature were comparable when restricted analytes were used (dry ice: 1.24 weeks (95% CI = 1.10 -1.37); ambient temperature 1.28 weeks (95% CI = 1.15-1.39). A similar pattern was also observed in terms of MAE (1.09 vs 1.12) (**Figure 4**). These values in fact were better than the published values from all analytes [17] RMSE 1.6 and MAE 1.24. There was a slight reduction in AUC (0.76, 95% CI = 0.68-0.84) compared to published ML algorithm [19] for samples when all analytes were included. However, the AUC remained similar when the ML algorithm with reduced number of analytes was used for 330 samples shipped in ambient temperature (0.73, 95% CI = 0.63-0.81) (**Figure 4**).

Evaluating the pattern of difference between prediction using all analytes and restricted analytes with the (Figure S2 in the **Online Supplementary Document**) main difference by restricting analytes was at the two extremes below 35 weeks and above 40 weeks. The minor shift to the left needs to be reviewed keeping in mind the sparsity of samples in these categories as well.

## DISCUSSION

In response to the recognition of the importance of accurate assessment of postnatal gestational age by the global health community, metabolic gestational dating approaches emerged in the last decade [13,42] to identify reliable methods for postnatal identification of gestational age. In LMICs settings with limited access to ultrasound dating, postnatal estimations can provide improved population surveillance to ultimately address issues of preterm birth prevention and to help target service delivery to high-risk preterm infants [43-45]. It can focus services necessary for improving outcomes, including kangaroo mother care and appropriate respiratory management and feeding [46]. In this study, we have demonstrated that machine learning algorithms developed using a sub-set of temperature insensitive, newborn screening analytes, are effective in deriving estimates of gestational age in infants born in the AMANHI cohort. The accuracy of the estimates being similar to published Iowa regression models using all analytes [17]. Indeed, we have shown that in using this subset of analytes and our models, the estimations of gestational age are identical between samples shipped on dry ice and those shipped in ambient temperature. Efforts are currently under way to begin implementing metabolic gestational age dating in low-resource settings to determine the burden of preterm birth and intrauterine growth restriction. Our finding could substantially facilitate the use of this method in settings of LMICs, where samples need to be shipped to referral centralized MS facilities, within or outside the country.

Concerns about the effect of temperature and time, on long term stability of metabolites [20,21] have been reported. Acylcarnitines have been shown to hydrolyze to free carnitines and corresponding fatty acids if stored for prolonged periods (>14 days) at room temperature [21]. Limited evidence exists regarding short term stability of amino acids and acylcarnitines in DBS. Stability with variations in temperature and time of 21 amino acids in DBS assessed by Han et al. [20], found Histidine most sensitive to temperature and Tryptophan sensitive to high humidity. Golbahar et al. [22] suggested the requirements of low humidity and temperature for transportation of dried blood spots. Adam et al. [47] have reported a loss of 4 markers with storage at 37°C. Typically in the developed countries, newborn metabolic screening is performed without any special temperature/humidity requirements. However, it needs appreciation that it is implemented in health facilities that already have ideal temperature/humidity control in place and most often the samples are processed within a few days at best. When this method is translated to LMICs the conditions change. The sample is to be collected after 24 hours of birth by which time the majority of mothers may have gone home and need to come to outpatient or the sample needs to be collected at home. Nearly all the health facilities catering to delivery of newborns do not have air conditioning and many times may have temperatures >38 degrees and humidity >70%. Finally, availability of tandem mass spectrometry facilities is limited and centralized; therefore, samples need to be stored and shipped to these facilities.

Three groups in North America have developed metabolic dating algorithms based on newborn health administrative data sets [17,48,49]. Research has since sought to validate these finding in LMIC settings where the application of these methods would be most useful. Hawken et al. [50] evaluated their algorithm across ethnic subgroups in Ontario. Murphy et al. [48] demonstrated external validation of the Ontario method in infants born in Bangladesh. We recently reported external validation of Iowa model in AMANHI cohort in Tanzania, Bangladesh and Pakistan [21]. We have since reported improvement in the accuracy of estimated gestational age using newborn screening analytes and our machine learning models developed within AMANHI cohorts [19].

Our study had several important strengths and some limitations. Strengths of our approach include a) the use of samples from a well-described cohort of infants with gestational age confirmed by first trimester ultrasound; b) availability of the parent external validity data from 1318 children in which our study of 330 paired samples was nested, enabling testing of the representativeness of the simulated training data set; c) masked, paired sampling design with results for each sample in the sub-set, available for both, shipment on dry ice and at ambient temperature enabling unbiased internal comparison in addition to comparison with parent study and published larger Iowa data set; d) availability of samples from both Asia and Africa with temperature, humidity and shipment conditions mimicking real life scenario; e) standard operating procedures (SOPs) for collection and storage of samples across sites enabling isolating impact of only shipment process. The primary limitation of this study is the small sample size. Although we tried to compensate by simulating a larger training data set and confirming simulated data set being representative of the parent data (Table S1 and Figure S1 in the **Online Supplementary Document**), potential limitations of specificity of our model to the population from which it was derived exists and the results need to be confirmed for external validity. Preliminary validation of our model in this sample with samples from both Asia and Africa, however, suggests robust performance across two important regions. Another limitation is the participation bias against very and extremely preterm infants, due to lack of survival of such infants in these settings as well as reluctance of parents for subjecting such newborns to these collection procedures. As a result, we had a relatively small number of samples collected from very preterm and extremely preterm infants, limiting our ability to comment on model performance in these sub-groups.

Our findings are encouraging but need further investigation. This work provides early evidence that gestational dating models developed using metabolic data from analytes resistant to temperature and humidity effects from Asian and African setting perform as well as the model originally published by our collaborators using data from a United States-born cohort of 230 000 infants using all available analytes in the metabolic screen. Overall prediction of gestational age was better with an average difference between predicted and actual gestation age of 1.12 weeks in ambient temperature restricted analyte model compared to 1.5 in Iowa model [17]. For differentiating between preterm (<37 weeks) and term (≥ 37 weeks) the model was marginally inferior with area under curve of 0.73 (95% CI 0.67-0.78) compared to Iowa [17] 0.90 (95% CI = 0.89-0.90) and AMANHI external validation [18] 0.86 (95% CI = 0.83-0.89). This seemed to be largely contributed by the shift in prediction of <34 weeks gestation to right and >40 weeks to left (Figure S2 in the **Online Supplementary Document**). It is difficult to tease from this sample whether this is a contribution of the sparsity of data in these gestation bins or actually a function of biological impact of elimination of some of the analytes that may be specifically associated. Further validation and investigation in a larger sample, especially a sample from a developed country with a larger proportion of newborns with gestation below 34 weeks is needed to address this issue. The evidence from testing the model comparing the samples shipped on dry ice (RMSE = 1.24, MAE = 1.09) and samples shipped in ambient temperature (RMSE = 1.28, MAE = 1.12) provides strength to this evidence (**Figure 4**). Thus, the trade-off between minor reduction in model accuracy with substantially reduced cost and flexibility in shipment and therefore implementation makes our method and models-based metabolic prediction models for gestational age highly suitable for most LMIC settings.

## CONCLUSIONS

Recently validated regression methods and machine learning approaches, to predict gestational age based on newborn screening markers, provide reasonably accurate postnatal assessments of gestational age, in settings where first trimester ultrasounds are limited. We have built upon our existing postnatal gestational age prediction machine learning models to demonstrate the predictive potential of using a limited set of temperature resistant newborn metabolic screening analytes. The value of these findings is 2-fold, first these models provide the feasibility of using this approach in low- and middle-income countries where the cost of shipment of samples may impede utilization of the approach; and second the approach provides a possibility for further investigation of identifying a suitable set of analytes that may be amenable to investigation by more broadly available autoanalyzer’s than less commonly available tandem mass spectrometry. Validation of our model in a larger sample is also warranted to determine its broader external validity and investigate its potential or lack thereof for identification of very preterm births.

## Additional material


Online Supplementary Document
