# Supplementary material for: Machine learning prediction of gestational age from metabolic screening markers resistant to ambient temperature transportation: Facilitating use of this technology in low resource settings of South Asia and East Africa
Source: J Glob Health. 2022 Apr 23;12:04021. doi: 10.7189/jogh.12.04021 (PMC9022771; doi:10.7189/jogh.12.04021)
Supplement: Online Supplementary Document [file jogh-12-04021-s001.pdf]

SUPPLEMENTARY INFORMATION

**Supplementary Figure 1: is a proper representation of the full dataset -80 storage and shipment (Sunil et al., 2021) with the 330 (bootstrapped to 10000) when stored and shipped in dry ice is terms of age group distribution**

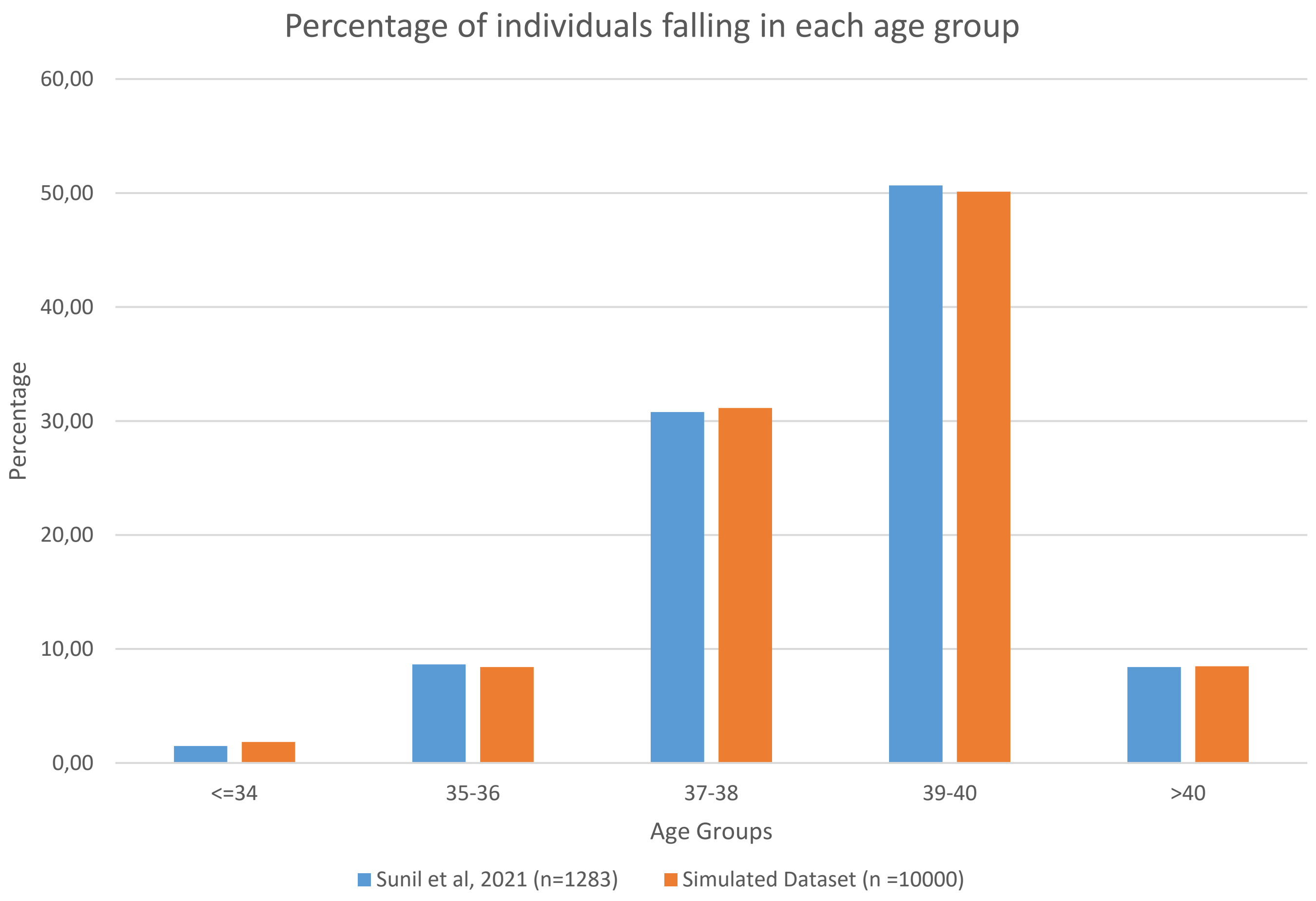

**Supplemental Figure 2: Comparison of predictions between samples in ambient temperature with those shipped in dry ice after elimination of temperature sensitive analytes**

| Age Groups | Actual GA | Selected_Dry | Selected_Ambient |
|---|---|---|---|
| < 34 | 13 | 0 | 0 |
| 35-36 | 23 | 18 | 18 |
| 37-38 | 103 | 175 | 173 |
| 39-40 | 165 | 133 | 135 |
| >40 | 26 | 4 | 4 |

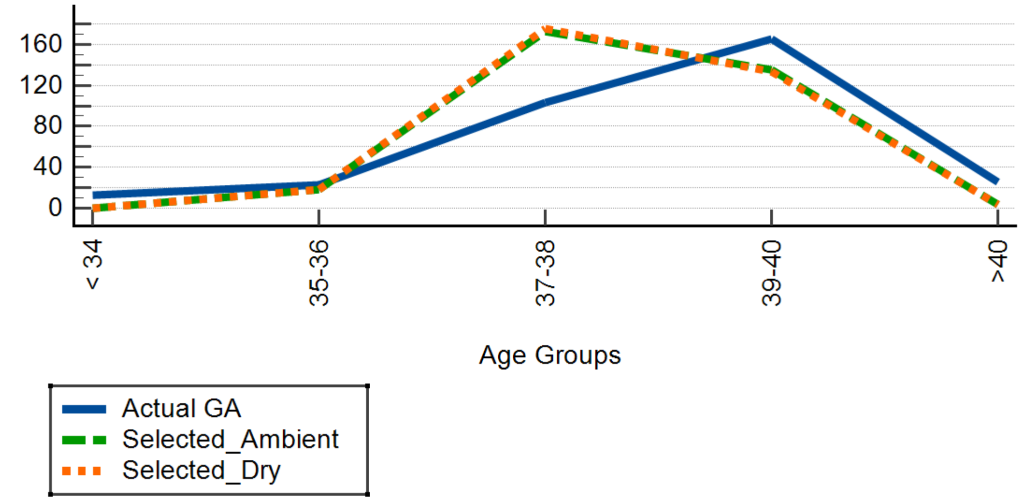

**Supplementary Table 1: Evaluating the representativeness of the simulated dataset from 330 new-born subsample with paired estimation to the overall 1283 new-born data and simulated dataset of 330 new-born subsample with reduced number of analytes**

| Dataset (-80 storage and shipment sample values) | RMSE | Bootstrapped 95 % CI | MAE | Bootstrapped 95 % CI |
|---|---|---|---|---|
| Full dataset -80 storage and shipment* | 1.02 | 0.91-1.14 | 0.76 | 0.65-0.88 |
| Simulated dataset from neonates with paired samples | 1.07 | 0.96-1.21 | 0.81 | 0.67-0.98 |
| Simulated dataset with reduced number of analytes from neonates with paired samples | 1.17 | 1.08-1.27 | 1.01 | 0.91-1.13 |

*Data published separately

**Supplementary Table 2: Analytes excluded* for prediction models with machine learning**

| Analyte Name | Percentage Change | p-value |
|---|---|---|
| 17 OHP | 20.42837 | 0.02337 |
| Ala | 40.20589 | 0.023062 |
| Arg | 44.78976 | 0.023667 |
| C10:1 | 26.79529 | 0.024215 |
| C2# | 35.74544 | 0.022767 |
| C3# | 28.6008 | 0.022767 |
| C5# | 25.24602 | 0.023255 |
| C5:1# | 31.7037 | 0.023588 |
| C6# | 32.403 | 0.023472 |
| Cit | 30.86818 | 0.021833 |
| Glu | 32.00174 | 0.021698 |
| Met | 30.536 | 0.021757 |
| Phe | 33.9038 | 0.021611 |
| Tyr | 29.50336 | 0.0213 |
| Val | 30.50249 | 0.0209 |
| Leu + Ileu | 29.61136 | 0.021833 |
| Bio1 | 25.10017 | 0.021733 |

***Exclusions were based on the literature review showing analytes amenable to variations with temperature and confirmation of the effect of temperature by paired comparison between results of dry ice and ambient temperature shipments.**
**All the analytes suggested in literature showed significant effect of temperature. In addition #analytes were excluded showing significant difference in our result**

**Supplementary Table 3: Metabolites used for the prediction of gestational age using machine learning**

| | |
|---|---|
| **Amino Acids** | Alanine (Ala), Arginine (Arg), Isoleucine + Leucine (Leu), Methionine (Met), Phenylalanine (Phe), Tyrosine (Tyr), Valine (Val) |
| **Acylcarnitines** | Acetylcarnitine (C2), Propionylcarnitine (C3), Butyrylcarnitine + Isobutyrylcarnitine (C4), Methylmalonylcarnitine (C4-DC), Isovalerylcarnitine + Methylbutyrylcarnitine (C5), Tiglylcarnitine (C5:1), 3 Hydroxyisovalerylcarnitine (C5-OH), Hexanoylcarnitine (C6), Octanoylcarnitine (C8), Octenoylcarnitine (C8:1), Decanoylcarnitine (C10), Decenoylcarnitine (C10:1), Dodecanoylcarnitine (C12), Dodecenoylcarnitine (C12:1), Tetradecanoylcarnitine (C14), Palmitoylcarnitine (C16), Palmitoleylcarnitine (C16:1), 3-Hydroxypalmitoylcarnitine (C16-OH), 3- Hydroxypalmitoleylcarnitine (C16:1-OH), Stearoylcarnitine (C18), Oleoylcarnitine (C18:1), Linoleoylcarnitine (C18:2) |
| **Enzymes & Hormones** | Galactose-1 Phosphate Uridyl Transferase (GALT), 17-Hydroxyprogesterone (17 OHP) |
